# The Best Day of the Week: New Technology Enhancing Quality of Life in a Care Home

**DOI:** 10.3390/ijerph16061000

**Published:** 2019-03-19

**Authors:** Anne Juul, Raelene Wilding, Loretta Baldassar

**Affiliations:** 1 Steno Diabetes Center Copenhagen, 2820 Gentofte, Denmark; anne.mette.juul.andersen@regionh.dk; 2 The University of Western Australia, Crawley, WA 6009, Australia; 3 La Trobe University, Bundoora, Melbourne, VIC 3086, Australia; r.wilding@latrobe.edu.au

**Keywords:** residential care, technology, social interaction, meaningful engagement, person-centred care, social ageing, social support

## Abstract

Older people living in residential aged care facilities tend to be physically as well as socially inactive, which leads to poorer health and reduced wellbeing. A lack of recognition of the importance of social support, limited resources, lack of training and task-oriented work routines leave little time for staff to meet the social needs of residents. Through qualitative ethnographic fieldwork, this study investigates the potential for new technologies to enhance quality of life and facilitate meaningful engagement in physical and social activities among culturally and linguistically diverse residents and staff in care facilities. A continuum from nonparticipation to full participation among residents was observed when Touch Screen Technology activities were implemented. Data indicate that resident’s engagement is impacted by five interdependent factors, including environmental, organisational, caregiver, patient, and management- &government-related. Findings show that new technologies can be used to increase meaningful physical and social engagement, including transcending language and cultural barriers. However, the successful application of new technologies to enhance quality of life is dependent on their integration into the daily routine and social relationships of staff and residents, with the full support of management. Guidelines governing the use of new technologies to support meaningful engagement of older people in residential care are lacking: this project highlights the importance of attention to the social relational dimensions of technology interventions to support best practice in their use.

## 1. Introduction

With the general trend towards people living longer and the growing proportion of adults over 65 years, the demand for long-term care is increasing, including community care, assisted living, long-stay hospitals and residential care [1,2]. Residential Aged Care Facilities (RACFs), also referred to as ‘nursing homes’ or ‘aged care homes’, are consistently associated with high degrees of social and physical inactivity, leading to boredom and loneliness [3,4,5]. In fact, RACF residents tend to be more lonely than community-dwelling older people, even though they are often surrounded by other residents and carers [6,7]. Limited resources, lack of training and task-oriented work procedures often leave little time for staff to meet the social needs of residents. Language and cultural barriers among and between care workers and residents further compound these challenges. This is significant in Australia, where approximately 1 in 3 people over the age of 65 were born overseas, mostly in non-English speaking countries [8].

Detailed evidence of activity in RACFs is provided by Den Ouden’s et al. [9] study of 723 nursing home residents in the Netherlands. Residents were randomly observed for one complete day, over a period of 16 hours. They reported that residents were inactive (sleeping, doing nothing and watching television) 45–77% of the time. In contrast, only 4–10% of observations recorded residents engaged in social interaction and communication activities. Similarly, Mallidou et al.’s [10] study of organisational resources, including staff time with residents in a long-term care facility in Canada, revealed that staff spend less than 1% of their time socialising. These high rates of inactivity and loneliness have negative impacts on the health, wellbeing and quality of life of residents in RACFs [11,12,13,14].

The ‘task-orientation’ of nursing care and the associated lack of time for social engagement is a concern commonly expressed in the sector [15,16,17,18]. In addition, staff are often not trained to engage residents in meaningful physical and social activities [19]. However, in order to improve successful ageing, RACFs need to be able to meet the resident’s physical and social needs. These concerns have given rise to a number of important responses, as well as a growing interest in meaningful engagement approaches including ‘Person-Centred Care’ (PCC) [20,21], which are gaining in popularity worldwide [22], and are supported by national standards such as the Australian Charter of Healthcare Rights [23]. There does not exist one specific meaningful engagement or PCC model, but rather a variation of different approaches [24].

Meaningful engagement approaches in residential care, including PCC, are associated with psychosocial benefits for residents as well as staff: staff experience a higher job satisfaction and improve their confidence and abilities to provide high-quality care [24] and the residents experience increased satisfaction with care as well as improved wellbeing [25]. The key aim of PCC, according to Edvardsson, Fetherstonhaugh and Nay [20] (p. 2614), is: “(…) *promoting a continuation of self and normality*”. Knowing the person, welcoming family, providing meaningful activities, being in a personalised environment and experiencing flexibility and continuity are all factors that contribute to PCC. Meaningful activities are an essential part of PCC and they improve the residents’ wellbeing, cognitive status and physical functioning [20,26]. In contrast, inactivity and lack of meaningful activities are found to have the opposite effect [27,28]. Slettebø et al.’s [29] Scandinavian study investigates how older people in residential care experience dignity, and demonstrates that activities need to be meaningful and individualised in order to foster self-worth and joy in residents’ daily lives. Active participation in meaningful activities can help residents to feel proud of being a member of the RACF community. In contrast, lack of meaningful activities renders care work less rewarding and more burdensome for staff and family members.

Alongside the emerging emphasis on meaningful engagement and PCC is a concurrent rapid growth in the use of new technologies to support older people’s wellbeing in RACFs. These include medical, assistive, information, communication and other everyday technologies [30,31,32,33]. Existing research focuses on how medical technology can assist staff in their work [34,35,36] or on how older people experience and benefit from the use of technology [37,38,39,40]. However, few studies investigate how technology can be used to facilitate physical activity and social interaction through meaningful activities in RACFs, and there are no guidelines to support their successful application.

This study is part of a broader research project on Ageing and New Media, which examines the role of social support networks in the health and wellbeing of older people and the importance of new media technologies in facilitating the exchange of care and support within these networks [41,42]. Our previous research on migrant and transnational families highlights the significant support role that ‘transnational’ and ‘distant’ family and friends can play in the wellbeing of older people. New communication technologies, like social media, allow them to stay in touch with their children and grandchildren, and to give and receive practical advice and support, including through ‘proxy’ arrangements facilitated by others [43,44]. More recent work has also examined the digital divide and ways to improve the digital literacy of older people, including through communities of practice models [45].

The aim of this study is to investigate the role of ‘Touchscreen Technology’ (TT) in facilitating increased physical activity and stimulating social interaction in RACFs in order to decrease social and physical inactivity. As part of our exploration of meaningful engagement, we are particularly interested in examining whether screen activities can be used autonomously by the residents or if they need to be directed and led by staff or volunteers. Social interaction and meaningful engagement are particularly challenging in RACFs that include ethnically diverse residents and care staff who speak a variety of languages. Hence, an additional question is whether touchscreen technology can facilitate activities across language divides. Our hypothesis is that TT activities might contribute to increased physical activity and social interaction, and thereby facilitate meaningful engagement to support improved physical health and social wellbeing of older people living in RACFs.

Existing literature suggests that social and physical inactivity in RACFs can be explained by a range of interdependent factors including: patient-related, such as immobility; organisational, such as lack of meaningful activities; environmental, concerning the facilities in the ward; and caregiver-related, including staffing levels, time constraints and type of care given [9,46]. In addition, we found that social and physical inactivity is to a large extent influenced by management- & government-related factors that shape and influence the other factors. The same set of factors also impacts on the successful integration of new technologies, particularly for initiatives focused on the social interaction and inclusion of residents.

## 2. Materials and Methods

### 2.1. Study Design and Methods

This study employed a case study design, which helps to understand a real-life phenomenon in a natural setting using multiple methods [47]. Data were gathered using a mix of qualitative ethnographic fieldwork tools:

We conducted participant observation in which we observed the residents’ behaviour in relation to the screen activities and participated by immersing ourselves in the group and the activity [48]. Field notes were taken at the end of the day using a protocol with descriptive as well as reflective entries [48] (*n* = 18).

We listened and engaged in targeted informal conversations with residents, staff and visitors (residents’ family and friends) about their experiences of the screen activities before, during and after the activity. These face-to-face conversations were conducted as informal interviews rather than formal interviews with structured questions. This method allowed us to document the participants’ immediate reactions and opinions about the activity. The conversations were recorded as a part of the field notes.

Video ethnography was used in the residents’ everyday setting in the common living room. This provided a distinctive contribution [49], which allowed us to observe the reactions of residents, staff and family members to the TT and explore the level of activity, participation and interaction with the TT when we were not present in the room.

In-depth semi-structured interviews were used in the broader research project on Ageing and New Media; residents (*n* = 15), family members (*n* = 10) and staff (*n* = 5) at this facility were interviewed. All interviews were approximately one hour in duration and were digitally recorded and transcribed. Interviews with residents included an exploration of their perceptions of growing older, their experience of retirement and their transition to living in residential care, with a particular focus on the impact this had on their social support networks. Interviews with family members and staff focused on their experiences of the residential care facility, as well as their perceptions of residents’ experiences of living there. These in-depth semi-structured interviews provide contextual information, including an understanding of how the RACF operates and prioritises activities and care.

### 2.2. Setting

The study was carried out in a residential aged care facility in Perth, Western Australia, hosting Culturally and Linguistic Diverse (CaLD) residents from non-English speaking backgrounds. Each resident has their own room, and they are located in three interconnected wards that each has a common living room and dining area. There are approximately 16 residents in each ward. The participants were residents from high care wards between 67–100 years old. Between 4–12 residents participated in the activity at a time.

### 2.3. Study Activities

According to Caprani et al. (2012), TT’s can be used in three different ways: (1) single user interaction with a device, e.g., smart phone; (2) multiple user interactions using the device at the same time; and (3) multiple user interaction with one device at different times [50]. TTs can be used to watch videos online, play games and for virtual social interaction.

The current study employs TTs in the second way, using a large 65″ (165 cm) interactive portable touch screen, with Windows software installed, on a mobile stand that could be rolled from room to room. It appeared to the residents like a large television.

An interactive physical activity video—Sitdance was uploaded and projected onto the screen. Sitdance is a seated dance tutorial specially designed for older people. According to the creator of the programme—Marcel Baaijens—Sitdance is not only an exercise programme, but the activity is also designed to support older people’s memory and encourages social interaction [51].

### 2.4. Data Collection

The data were collected over the course of 10 months when the researchers, Juul and Baldassar, visited the different wards approximately once a week, on the same day of the week, for a total of 18 visits.

A group of residents would typically be in the living room watching television when we arrived. We would ask them whether we could turn off the television and put on the TT screen instead.

The way we ran the activity was adjusted over the course of our visits as a result of observations and reflections. As we were interested in investigating whether the participation level depended on the presence of an instructor that lead the activity, we organised the activity in three different ways to facilitate a comparison of participation levels:

(1) In the first set of weekly visits we set-up the Sitdance activity and participated in it as leaders, standing at the front of the room.
(2) In the second set of weekly visits, after setting up the screen and starting the Sitdance activity, we limited our involvement to observation only during the first three songs and then participated in the last three songs to record any difference in participation levels. We noticed that even if we were standing passively at the back of the room, residents would continue to look for us.
(3) In the third set of weekly visits, we set-up the Sitdance activity and then left the room, using video ethnography to record the response to the activity in the absence of the researchers. Then we repeated the format of the second set of visits.
(4) In the final set of weekly visits, we repeated the variations of the previous three sets of visits: leading the activity, being present but not participating, setting up the activity and leaving the room.

In addition, we also varied the level of leadership and type of interaction with participants during the activity including leading from the front of the room by doing the movements as instructed in the video and singing; sitting beside residents in the room while doing the movements and singing; and motivating and encouraging resident to participate verbally and with body language by smiling and nodding and talking. Lastly, we physically assisted the residents with their movements.

### 2.5. Data Analysis

Initially all field notes and video footage were thoroughly reviewed to identify main themes [52]. Next, the data were coded to refine themes and select representative quotes and field notes to illustrate each theme [48]. As observation was an ongoing process, the analysis had cyclical features [53].

### 2.6. Ethical Considerations

The project obtained ethical approval from the University of Western Australia Human Research Ethics Committee (Reference Number RA/4/1/8132) and consent was obtained from residents or their family members, as well as the care facility. All resident and staff names and private information are anonymised.

## 3. Results

Analysis of data identified three interdependent key themes concerning social and physical interaction in the facility: (1) Social Interaction in RACF: Everyday Inactivity—‘Just Sitting Here; (2) Initiating Activities with TTs: ‘It’s Not a Magic Pill; and (3) Ambivalence Among Staff—‘Is This Something We Should Be Doing?’’ The quotes included below are extracts from field notes in which informal conversations were recorded.

### 3.1. Social Interaction in RACF: Everyday Inactivity—‘Just Sitting Here’

During the observations at the RACF, we observed that the residents’ daily routine mostly involved sitting passively and ‘watching’ television in the common room, apart from time spent being dressed, fed, toileted, or given medication. In the course of our participant observation, residents commonly commented on the lack of activities, stating, for example, that, “*we are just sitting here*” and they expressed a strong desire to be, “*doing something*”. Residents quickly became accustomed to our weekly visit and TT activity, which elicited positive comments like, “I have seen this screen twice”, “*I have been waiting*” and, “*we should do it more often*”. During informal conversations after the activity one woman told us that, “*the best day of the week*” was when we came to do the Sitdance on the TT in the common room.

It was not only the residents that expressed a sense of disappointment over the lack of activities. This was also commonly noted by family members, exemplified in the following field note entry:

“*Three daughters were visiting their mother. They were taking their mother out of the room when we came into the room with the screen. One of the daughters stopped and came to ask what we were doing. When they heard about the activity they decided to roll the mother’s wheel chair back into the room. (…) During the activity, they told us how frustrated they are with the lack of activity: ‘they are just sitting in front of that telly all day*’.”

When family and visitors were told about the activity, they were generally very curious and enthusiastic about social interventions of any kind.

Staff members also expressed concern about inactivity, making comments such as: “*Now they are doing something, they enjoy doing something*”. These statements show that staff and family members understanding of the activity level is consistent with the residents’ experiences: the residents are not doing much social or physical activity in a normal week day.

### 3.2. Initiating Activities with TTs: ‘It’s Not a Magic Pill’

We found that touch screen activities, like Sitdance, can be a tool to initiate physical activity and social interaction in the RACF. When the screens were set up and the activities were on, there was a very clear shift in the mood in the room. While watching television is a passive and sedentary activity, the TT activity gave residents the opportunity to be a part of a social activity, as well as providing mental and physical stimulation.

The TT activity increased both physical and social interaction between the residents: they looked and smiled at each other and, at times, they ‘danced’ together with each other, exemplified in the following field note:

“*Now she* [a resident] *reaches for another resident’s hand. For a while, the two residents are dancing together [while seated]. One of them, the hesitant one, has limited English abilities, the other one only says the same three words in Macedonian. But no matter the physical, mental and language abilities, the two women interact during Sitdance*.”

In addition, the activity initiated interaction between residents and their family members. The field note extract below describes the visit of Eleanora, one of the resident’s daughters:

“*Eleanora has been participating many times and enjoys the activity. The daughter participates in the activity and the mother and daughter often look each other in the eyes during the activity. After the activity I [the researcher AJ] talked with the participants: I say ‘goodbye’ to Eleanora and her daughter. I say to the daughter: ‘thank you for also participating’, to which she answered: ‘Are you kidding me, I loved it, and I could see how mom loved it too—that is why I stayed*’.”

In contrast, it appears that family members who are present and choose not to participate hinder the participation of residents, which the following observation illustrates:

“*The daughter of one of the residents is here today. When I enter the room, she asks me what I am doing and I explain the activity to her. ‘Oh, that’s nice’ she says. She leaves the room. Her mother starts to participate. Her mother is quite mobile and can move both arms and feet by herself. During the second song, the residents’ daughter comes back, and the resident stops participating. The daughter is sitting and talking to the mother. It is quite distracting for the rest of the residents. She talks loudly because of the music. During the third song, the daughter leaves again, and the mother starts participating again. When the daughter re-enters during the 5th song, she begins to lecture her mother; (…) ‘and mom, you know, it is good for you to move, or else one day you cannot move by yourself anymore*’.”

We recorded just three instances of family members who were present for the activity but chose not to participate. It is possible that these family members, who were all relatively old themselves, may have feared being identified as residents had they participated. For example, one man laughed nervously when we encouraged him to participate saying, “*oh no, I am just visiting today”*.

The residents’ level of participation varied depending on their physical and mental abilities. Whereas some were able to follow the activity as shown on the screen, moving their bodies to follow the directions in time with the music, others only tapped with their foot or moved their head to the rhythm, some sang and others just smiled and actively observed. Thus, TT can facilitate inclusive activities that allow residents to participate in accordance with their desires and abilities.

The RACF we visited hosts many CaLD residents and has a very culturally and linguistically diverse staff. All the staff members spoke English to varying degrees of ability, but some of the residents had very limited English skills. Despite this, the Sitdance activity allowed the residents and staff to have a social interaction through nonverbal interaction, as was evident in this encounter:

“*Rosa, who has been in the room at least 2–3 times without participating sits next to a carer. She does not speak much English. The carer smiles and laughs a lot. She encourages Rosa to participate by showing her how to do it, taking her hands and looking her in the eyes. She also uses verbal encouragements. The woman starts participating and smiles throughout the remainder of the session*.”

This interaction illustrates how activities using TT have the potential to transcend language and cultural barriers. A continuum from nonparticipation to full participation was observed. The extent of participation was influenced by the presence of leaders and degree of leadership. When no one else other than the residents were present, the activity level was limited and those who participated did not use their full ability. The following two field notes illustrate how the same woman’s activity level changed, depending on the presence of a leader. Commenting on Maria’s participation in the video, when the researchers are not present:

“*In the middle of the song, Maria, who is sitting in the right-hand corner, starts to move her head a little bit to the rhythm of the music. She is looking up and down as instructed, but her movements are very small. At one point, she is carefully moving one of her legs. She is now counting the rhythm: ‘One, two, three and four’. She is no longer moving her head up and down but is only nodding to the rhythm*.”

When we led the activity, while motivating and encouraging the residents to participate, Maria’s activity level increased remarkably: “*Maria, who is sitting in the right-hand corner, is singing* [the verse in the song]*, while she is doing the exercise. She is particularly active and able to do the exercises very well*.” This finding is indicative and shows that the activity is considerably more effective when led. Our observations also suggest that the touch screens are not used autonomously by the residents.

Moreover, the observations showed that the degree of leadership matters. When the resident was instructed, verbally praised and encouraged to participate, the participation level increased. The participation level further increased when the residents’ additionally received physical support from carers, as described in the following field note: “*A staff member went behind the two residents, one by one, held their hands, leading their hands to the rhythm of the music and helped them to do the exercises. When the carer stopped, they continued for a while*.” Here the participation level changed from passively looking and smiling to physical participation, which they visibly enjoyed.

When staff facilitated the participation of residents, they reported having a positive social interaction with the resident. Our observations also showed that the residents often directed their attention to the leader of the activity as opposed to the screen. This emphasises how the TT is an instrument that can be used to assist social interaction and activities at RACFs, but it should not be considered a ‘magic pill’ that can initiate activities without facilitation. The contact and social interaction with the staff/leader is integral to the activity, indicating the importance of social-relational dimensions to the successful application of new technologies for meaningful engagement outcomes.

### 3.3. Ambivalence Among Staff—‘Is This Something We Should Be Doing?’

As is common across the sector, it became clear during our observations that the staff worked on a task-oriented timetable, which focused on the physical needs of residents. A standard daily routine was described to us: “*getting them up in the morning, dressing them, feeding them breakfast, giving morning tea, then lunch, then afternoon tea, then dinner, then helping them to bed, as well as taking them to the toilet maybe 5 times a day*.” Further discussions with management revealed that the funding model governing staff care does not include social care, which is either not considered important or is expected to happen ‘naturally’. In spite of this, most of the staff were very attentive and responsive to residents’ moods, shown in particular by their use of nonverbal touch. We observed, for example, that staff had the potential to have a great impact on the participation level and, thereby, increase the physical and social activity of residents. When staff spontaneously participated in the TT activity, they assisted the residents to move, they smiled, laughed and clearly enjoyed the activity. This had an overall positive impact on the mood of the room and increased the interaction between everyone.

Nevertheless, every time we encouraged and invited the staff to use the screen and offered to show them how, they immediately showed reluctance. They did not want to take responsibility for learning how to use the screen or conducting the activity. They explained that they needed to prioritise the resident’s physical needs and that they experienced time and job constraints. They were also unsure whether management would view their engagement with the screens as the best use of their time. Moreover, lack of confidence using technology was also experienced as a significant challenge. Thus, to utilise the benefits of the touch screen activities, the staff require proper training and, perhaps most importantly, full and explicit support from management, including the integration of technology interactions into their work schedules.

## 4. Discussion

**Figure 1:**
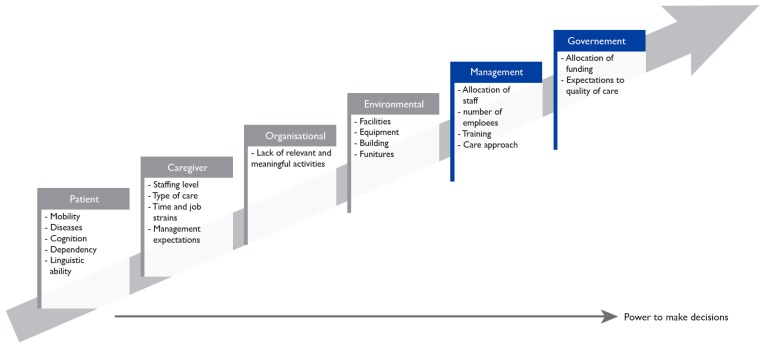
Factors influencing residents’ physical and social inactivity in residential care extending the work from Douma et al. [46] and den Ouden et al. [9].

The overall purpose of this study was to examine if and how TT could be used to increase physical and social activities at a RACF in order to reduce inactivity and facilitate interaction across language barriers. Additionally, we wanted to shed light on whether the touch screen activities needed to be directed and led, or if they could be used autonomously by residents. As noted earlier, a number of factors shape the experience of physical and social (in)activity among residents in an RACF. In this section, we discuss how these factors impact upon the successful integration of TT activities in facilitating meaningful social and physical engagement, including environmental, organisational, patient, caregiver and management- & government-related factors, see Figure 1.

### 4.1. Environmental Factors

Environmental factors concern the facilities in the ward that make it possible for residents to engage in such activities as walking around the ward or residence, setting the table, or performing other household activities [9] (p. 5). When we first entered the RACF, we immediately noticed its pleasant and welcoming environment. The residents have private rooms linked to large, bright and open hallways with easy access to spacious, open common living rooms. This design allows staff and visitors passing through to interact with residents and facilitates opportunities for engaging in different activities. In addition, residents were able to walk around the wards and the physical environment and facilities provided the potential for social and physical activities, which is in accordance with den Ouden et al.’s findings [9]. This is important, as the physical environment is central to residents’ wellbeing, when utilised well [54,55]. However, due to a combination of organisational-, patient-, caregiver-related factors and management- & government-related factors, the potential of the environment was not fully utilised.

### 4.2. Organisational Factors

While residents, their family members and staff were all very positive about the level of medical and physical care provided by the facility, our data indicate that they share a sense of disappointment over the general lack of meaningful physical and social activities for residents, who spend several hours each day passively sitting in front of the television. It is not unusual for RACF residents to suffer from boredom and loneliness as a result of lack of meaningful activities [56,57]. Lack of meaningful activities can be defined as an organisational factor that contributes to inactivity [9] (p. 5).

The TT Sitdance activity provided an opportunity for residents, their family members and staff to engage in a meaningful activity in several ways. Firstly, it increased their physical activity level, encouraging body movement, including mental stimulation required to follow the instructions with hand–eye coordination and keep in time to the music. Secondly, it provided an opportunity to engage socially with each other, observing and commenting on the shared experience, often inspiring singing together and triggering the sharing of memories. For example, many participants reported that they had regularly enjoyed dancing before entering the facility, but that they were no longer able to, and the Sitdance activity was, “*like dancing again*”. Thirdly, it lifted the mood of the room, transforming it into a livelier space, facilitating a sense of community and shared fellowship among all participants. Through the activity, residents went from being individuals doing nothing to being active members of a group, contributing to a sense of belonging.

In this study we only used the TT for the Sitdance activity, and thus our results cannot necessarily be generalised to other activities. However, one of the advantages of TT is that it can be used for a broad variety of activities and not only Sitdance. This makes it possible to personalise the activities to meet the needs of a specific resident or group of residents with shared interests. For example, games or drawing apps could be introduced, or memory and reminiscence activities could be stimulated, for example, by virtual visits using map applications. Reminiscence activities have been demonstrated to reduce loneliness, facilitate opportunities for meaningful activities and increase wellbeing [58,59,60,61]. Thus, TT has the potential to reduce inactivity by providing meaningful activities at RACFs to help combat boredom and loneliness and to support meaning engagement and PCC approaches in nursing. However, to be effective, other factors must be addressed.

### 4.3. Patient-Related Factors

Different patient-related factors, such as care dependency, mobility problems, chronic diseases, fatigue, cognition and habitual inactivity of the resident [9] (p. 5), likely influenced the high levels of inactivity and sedentary positions observed. Like most other OECD countries, Australia’s aged care policy is focused on ‘ageing in place’, that is, supporting older people to remain living in their own home instead of moving into residential care [62,63,64]. As a result, most people who move into residential care have high care needs [8,65]. Most of the residents who participated in our study had a high degree of physical disability, thus, the inactivity in the RACF was significantly patient-related. However, limited mobility does not necessarily cause social and physical inactivity, depending on whether suitable activities or supports are identified and provided. Individual physical therapy was provided to residents daily, however group activities were limited.

Our data clearly show a continuum from nonparticipation to full participation depending on the presence of a leader and the degree of leadership, verbal encouragement and physical support. The more mobile residents were able to fully participate in the Sitdance activity, whereas the less mobile residents participated by nodding, singing, smiling, having eye contact and moving as much as their physical condition allowed. It was clear that the highest participation level was reached when residents were verbally encouraged and physically supported by staff or the researchers. The vast majority of residents, regardless of their physical capacities, enjoyed the opportunity for nonverbal communication through touch, for example, holding their hands/arms/feet to move them in time with the music.

Touching has always supported verbal communication in nursing. Touching can roughly be divided into ‘necessary touch’, that is, when the touching has a functional purpose, for example nursing tasks, and ‘unnecessary touch’, which is a touch that is used to show affection, care or attention [66], also called ‘caring touch’ [67]. We would argue that ‘caring touch’ is necessary for social wellbeing, particularly for those residents who are unable to speak. We used caring touch to support resident’s participation in the activity and we encouraged staff and family members to do the same. We always asked residents’ permission before touching them, and sought verbal as well as nonverbal cues of acceptance, in accordance with intimate touch recommendations [68].

Not surprisingly, higher participation levels, particularly for residents with physical disability, was associated with leading the activity and supporting resident participation through verbal and nonverbal encouragement and touch. However, even the more mobile residents showed a marked increase in participation level with leadership. This finding suggests that residents are primarily motivated to participate by and for the social and emotional interaction, rather than for the opportunity to be physically active. This leads us to argue that when residents say they want ‘to do something’, they are likely also indicating that they want to feel (or that they lack feeling) socially engaged and a sense of belonging. Thus, TT is most effective when the activity is led and directed to facilitate social engagement.

Social relationships at RACFs are essential for residents’ quality of life [27,69]. A study exploring ways residents develop relationships at RACFs reveals that social relationships are largely an unintended consequence of trying to have a life through participating in daily activities [70]. Researchers reported that residents participate in activities, not because they see an inherit value in the activity, but because it is an opportunity to engage in a social relationship. This indicates very clearly that efforts to increase physical activity at RACFs will be more successful if they include a strong social relational dimension. Furthermore, if the social dimension is not carefully accounted for, then the use of technology in RACFs may lead to reduced human contact, as reported by Cahill, McLoughin and Wetherall [71]. Our findings show that using TTs with the explicit aim of increasing physical and social engagement can increase the key dimensions in person-centred care, including eye contact and touch, among residents and between residents and staff.

Finally, in addition to transcending physical immobility capacities, the TT activity can transcend language barriers. It is not unusual to see a decline in older migrant’s linguistic abilities [72] and many of the residents in our study tended to use their first language, which was not English. While many of the staff members speak several languages, they were often not able to communicate with residents beyond very basic interactions, which can impede care beyond the most basic physical necessities. This emphasises the need for appropriate activities that can stimulate interaction between staff and residents that transcend the need for language. TTs offer this potential to support meaningful social and physical activities with CaLD persons in residential care, facilitating communication across language differences regardless of physical and mental capacities.

TTs show great potential to support meaningful activities in residential care but these activities need to be led and supported by verbal and nonverbal encouragement and support, and designedto facilitate social engagement. This has important staff and resourcing implications.

### 4.4. Caregiver-Related Factors

Given the high care needs of RACF residents, it can be argued that their social and physical activity levels are largely determined by caregiver-related factors, such as staffing levels, time constraints and types of care provided [46] (p. 5). Our data show that staff activities were largely characterised by a medical and physical task-oriented approach, which does not easily foster time and attention to initiating and participating in social activities. These findings are supported by recent studies by Den Ouden et al. [9] and Douma et al. [46], which indicate a need for increased opportunities for social interactions at RACFs. Staff experience of time and job constraints can affect the degree of person-centred care [73]. Sjögren et al. [74] investigated the relationship between RACF staff perceived work environment and person-centred care, and found that higher levels of staff satisfaction and lower levels of job strain make it possible for staff to interact and initiate activities that are in accordance with the residents’ preferences and needs [74]. Our study explored how TTs may provide this kind of opportunity.

There is a growing body of literature that shows how technology can be used as an enabler to connect with others [75]. However, a systematic literature review investigating the impact of technology on older adults’ social isolation shows that most of these studies focus on general Information and Communication Technologies (ICTs) and Social Network Sites (SNS) [76]. As most RACF high care-residents have limited physical and mental capacities, such interventions are not always suitable. Hence, this study investigated whether TT can facilitate meaningful activities to reduce physical and social inactivity. Our results show that TT has the potential to be used as a tool to initiate social interactions between residents, as well as between residents and staff, and family and friends. The screens are used as a tool to initiate social contact within the RACF. However, as we have shown above, to be successful, attention must be given to the importance of social relational dimensions of technology interventions. Thus, the TT activities need to be integrated into the daily routine and social relationships of staff and residents.

In addition to time and job constraints, staff reported a lack of knowledge and lack of confidence using the TT. They were also ambivalent about whether using the TT was an effective use of their limited time and whether it would be supported by management. Goh et al. [77] investigated RACF staff views and perceived barriers to TT use to engage residents with dementia in meaningful activities and found that an education session can help the staff to be more self-confident in their abilities to use TT with the residents. After presenting our findings at a staff meeting, with full management support, we did notice an increase in positive attitudes among staff towards the use of the screens. However, they were only actually used independently by staff when formally scheduled into work tasks. Other research reports that RACF staff need more formal training, including in services and step-by-step demonstrations [78], in order to increase staff confidence and ability using new technologies.

Given the resourcing and time constraints on staff, the management in our study were keen for the TT activities to be taken up by family members and volunteers. While this is a positive endorsement and recognition of the value of TTs in supporting meaningful activities, it is debatable whether it goes far enough. Social support and positive relationships with staff are related to quality of life and thriving in residential care [54,79,80]. A qualitative study from Norway of resident’s experience with interpersonal factors at RACFs found that quality nursing entails: “(…) *a balanced, individual approach to medical, physical and psychosocial care, including interpersonal aspects of care*” [81] (p. 1365). Accordingly, it can be argued that social support from others, such as volunteers, would not yield the same benefits as when the staff are responsible to ensure the residents’ need for social interaction are met. For this to occur, staff require adequate training, not only in the use of new technologies, but in recognising how social support is associated with health and wellbeing benefits. In addition, management needs to endorse and support staff to learn these skills and value this work.

### 4.5. Management- & Government-Related Factors

In addition to the four factors identified by Douma et al. [46] and den Ouden et al. [9] and discussed above, we found that the residents’ inactivity is related strongly to management- & government-related factors. To our knowledge, this constitutes an under-examined factor when investigating the cause of older people’s physical and social inactivity at RACFs.

In our study it was evident that the staff played a major role in the residents’ physical and social activity. Without interventions from staff, residents spend most of their day passive and sedentary. However, the caregivers cannot implement TT-based activities into their work schedules without management approval. This is in-line with Sjögrens et al.’s [82] conclusion in their study investigating person-centred care characteristics, that it is essential that managers and leaders in residential care enable staff to prioritise social interactions and encounters. Thus, management needs to fully endorse the use of new technologies by staff, including providing permission, education and training as well as clear directions.

The management staff in this study were very responsive and aware of the problems with residents’ physical and social inactivity. Even though they wanted to make improvements, they were financially challenged because of limitations in funding. RACF funding and work schedules focus on physical and medical care and do not include resources for meaningful activities that support the resident’s social wellbeing. The government assesses the residents’ relative care needs using the Aged Care Funding Instrument (ACFI) and allocates funding on this basis. It consists of 12 questions divided into three domains: Activities of Daily Living Domain regarding nutrition, mobility, personal hygiene, toileting and continence; Behaviour Domain related to cognitive skills, wandering, verbal behaviour, physical behaviour and depression; and Complex Health Care related to medication and complex health care [83]. However, this instrument inadequately assesses the residents’ social and emotional needs, including a lack of recognition of the importance of social support [84]. In a recent report on Alternative Aged Care Assessment, Classification System and Funding Models in Australia, Mcnamee et al. [84] (p. 23) argue: “*Engaging residents in meaningful activities and promoting participation and independence in an ongoing way (i.e., the wellness approach) should be regarded as the care standard for all residents in aged care settings who are likely to benefit. Provision of a wellness approach to care should be reflected in the Accreditation Standards*.” Nevertheless, there is a tendency to view social and physical activities as an optional extra or to view this type of activity as something that ‘just happens naturally’ without formal planning. In our research experience, this is rarely the case in practice.

## 5. Conclusions

Ultimately, residents’ physical and social activity levels are impacted by a number of interdependent factors that are environment-, organisation-, patient-, caregiver-, management- &government-related, as illustrated in the figure above. If the government does not formally recognise the value of meaningful social and physical activities, management do not have the resources to support them (management- & government-related factors). If management do not provide an appropriate environment with access to the equipment needed, staff do not initiate social activities (environmental factor). If the staff do not initiate appropriate and meaningful activities that take the residents’ physical and mental abilities into account (organisational and caregiver-related factors), the residents remain physically and socially inactive. Residents’ physical and mental capacities limit their ability to be socially and physically active (patient-related factors). However, they are by no means the only relevant factors.

Some will question whether TTs offer any advances on other tools that might be used to achieve the same goal, such as having a volunteer or trained staff member delivering Sitdance in person rather than on a screen, or delivering another activity such as painting or gardening. We are not suggesting that technology is a better tool, rather, that it is a potentially useful tool if used appropriately. Our participant observation over 10 months at a modern aged care facility showed that even if the management and staff have the best intentions, meeting the needs of individuals from a variety of cultural backgrounds is not easy. There is an increasing demand for high quality, individualised care, but, as we have shown, time constraints and other factors make it difficult to meet resident’s needs [85]. As Boyd et al. accentuate in their article, ‘Bored to Death—tackling lack of activity in care homes’ regarding older people with dementia: “*Many people with dementia will spend the day clean and well-fed, but unstimulated and inactive*” [57] (p. 98), which corresponds with our observations. The use of technology not only provides residents with instrumental and functional support, but it has the potential to stimulate social interaction with each other and with staff, and can help transcend language barriers. This emphasises the social engagement potential of introducing new technologies into RACFS.

Our aim is not to suggest that TT is the only or necessarily the best solution to the inactivity of residents at RACFS. Rather, in a time when increasing demand for high quality, long-term care coincides with limited resources and task-oriented work regimes, we suggest that TT can offer an opportunity to increase the activity level and opportunities for social interaction, and thereby increase the quality of life, of residents.

A growing proportion of older adults are bound to increase pressure on the aged care sector. RACFs need to provide not only high-quality care, but also care that is cost-effective. Our study shows that technology can help to deliver a much-needed resource-efficient solution to social and physical inactivity in aged care. The TT can be used to decrease social and physical inactivity by initiating meaningful activities in accordance with the residents’ values, preferences and physical, social and linguistic abilities, and thus has the potential to reduce social and physical inactivity.

While modest, our results indicate that these interventions do not necessarily need to take a lot of time; we conducted the TT activity once a week, spending approximately 30 minutes in each ward. However, major challenges to the successful use of TT include staff time and job strains, lack of relevant training in TTs and management support. Implementing TTs at RACFs would require restructuring staff duties and tasks to include meaningful social and physical activities. There is an ongoing debate about whether it is realistic to expect staff to have the time to increase the physical and social activity of residents. This is not necessarily a matter for individual institutions to address, as they are constrained by the government funding models within which they operate. Thus, it is a matter for policy makers to question whether incorporating social activities is a worthwhile investment of limited resources. At the same time, we would question whether the sector can afford not to. Physical and social inactivity is not only related to adverse health benefits, it is also associated with poorer wellbeing.

Our results provide some recommendations for practice, in particular, the need to acknowledge the role of social support and interaction in the wellbeing of RACF residents, and the importance of recognising the social relational dimensions of technology interventions to support their successful application. Government and policy-makers should recognise the importance of increasing physical and social activity and prioritise financial support for technology and training that can assist RACFs with this. Future research should investigate the effectiveness of TT staff training on residents in these areas.
